# Performance of the inFLUenza Patient-Reported Outcome (FLU-PRO) diary in patients with influenza-like illness (ILI)

**DOI:** 10.1371/journal.pone.0194180

**Published:** 2018-03-22

**Authors:** John H. Powers, Elizabeth D. Bacci, Nancy K. Leidy, Jiat-Ling Poon, Sonja Stringer, Matthew J. Memoli, Alison Han, Mary P. Fairchok, Christian Coles, Jackie Owens, Wei-Ju Chen, John C. Arnold, Patrick J. Danaher, Tahaniyat Lalani, Timothy H. Burgess, Eugene V. Millar, Michelande Ridore, Andrés Hernández, Patricia Rodríguez-Zulueta, Hilda Ortega-Gallegos, Arturo Galindo-Fraga, Guillermo M. Ruiz-Palacios, Sarah Pett, William Fischer, Daniel Gillor, Laura Moreno Macias, Anna DuVal, Richard Rothman, Andrea Dugas, M. Lourdes Guerrero

**Affiliations:** 1 Clinical Research Directorate/Clinical Monitoring Research Program, Leidos Biomedical Research, Inc., NCI Campus at Frederick, Frederick, MD, United States of America; 2 Evidera Evidence, Value & Access by PPD, Seattle, WA, United States of America; 3 Evidera Evidence, Value & Access by PPD, Bethesda, MD, United States of America; 4 National Institutes of Allergy and Infectious Diseases, NIH, Bethesda, MD, United States of America; 5 Madigan Army Medical Center, Fort Lewis, WA, United States of America; 6 Infectious Disease Clinical Research Program, Department of Preventive Medicine and Biostatistics, Uniformed Services University, Bethesda, MD, United States of America; 7 Henry M. Jackson Foundation for the Advancement of Military Medicine, Rockville, MD, United States of America; 8 Naval Medical Center, San Diego, CA, United States of America; 9 Defense Institute for Medical Operations, San Antonio, TX, United States of America; 10 Naval Medical Center, Portsmouth, VA, United States of America; 11 Walter Reed National Military Medical Center, Bethesda, MD, United States of America; 12 Children’s National Medical Center, Washington, DC, United States of America; 13 Instituto Nacional de Enfermedades Infecciosas, Mexico City, Mexico; 14 Hospital General Dr. Manuel Gea González, Mexico City, Mexico; 15 Instituto Nacional de Ciencias Médicas y Nutrición Salvador Zubirán, Mexico City, Mexico; 16 UCL, London, United Kingdom; 17 INSIGHT Influenza Study Groups, Rochester, MN, United States of America; 18 Institute of Clinical Trials and Methodology, University College, London, United Kingdom; 19 Institute for Global Health, University College, London, United Kingdom; 20 Kirby Institute, University of New South Wales, Kensington, Australia; 21 University of North Carolina, Chapel Hill, NC, United States of America; 22 University of Cologne, Cologne, Germany; 23 Hospital General de Agudos JM Ramos Mejia, Buenos Aires, Argentina; 24 Johns Hopkins University School of Medicine, Baltimore, MD, United States of America; University of Washington, UNITED STATES

## Abstract

**Background:**

The inFLUenza Patient Reported Outcome (FLU-PRO) measure is a daily diary assessing signs/symptoms of influenza across six body systems: Nose, Throat, Eyes, Chest/Respiratory, Gastrointestinal, Body/Systemic, developed and tested in adults with influenza.

**Objectives:**

This study tested the reliability, validity, and responsiveness of FLU-PRO scores in adults with influenza-like illness (ILI).

**Methods:**

Data from the prospective, observational study used to develop and test the FLU-PRO in influenza virus positive patients were analyzed. Adults (≥18 years) presenting with influenza symptoms in outpatient settings in the US, UK, Mexico, and South America were enrolled, tested for influenza virus, and asked to complete the 37-item draft FLU-PRO daily for up to 14-days. Analyses were performed on data from patients testing negative. Reliability of the final, 32-item FLU-PRO was estimated using Cronbach’s alpha (α; Day 1) and intraclass correlation coefficients (ICC; 2-day reproducibility). Convergent and known-groups validity were assessed using patient global assessments of influenza severity (PGA). Patient report of return to usual health was used to assess responsiveness (Day 1–7).

**Results:**

The analytical sample included 220 ILI patients (mean age = 39.3, 64.1% female, 88.6% white). Sixty-one (28%) were hospitalized at some point in their illness. Internal consistency reliability (α) of FLU-PRO Total score was 0.90 and ranged from 0.72–0.86 for domain scores. Reproducibility (Day 1–2) was 0.64 for Total, ranging from 0.46–0.78 for domain scores. Day 1 FLU-PRO scores correlated (≥0.30) with the PGA (except Gastrointestinal) and were significantly different across PGA severity groups (Total: F = 81.7, p<0.001; subscales: F = 6.9–62.2; p<0.01). Mean score improvements Day 1–7 were significantly greater in patients reporting return to usual health compared with those who did not (p<0.05, Total and subscales, except Gastrointestinal and Eyes).

**Conclusions:**

Results suggest FLU-PRO scores are reliable, valid, and responsive in adults with influenza-like illness.

## Introduction

Influenza (flu) is characterized by an array of symptoms, including chills, cough, sore throat, runny or stuffy nose, fatigue, muscle/body aches, and potentially diarrhea and vomiting, with symptoms ranging in severity and duration [1]. In the absence of known influenza virus, this constellation of symptoms can be caused by a variety of other viruses and is often diagnosed as influenza-like illness (ILI) [2]. While most patients recover from ILI, the symptoms can negatively impact daily activities and functioning. Symptoms of ILI often closely approximate influenza symptoms such that the two previously have been indistinguishable. Therefore, a symptom measure useful in influenza may also be useful for evaluating the presence and severity of symptoms of ILI.

The InFLUenza Patient-Reported Outcome (FLU-PRO) measure was designed to evaluate the presence, severity, and duration of influenza symptoms in clinical trials. Developed using good research practices for scale development methods [3–5], including those recommended by the US Food and Drug Administration (FDA) [6], this 32-item daily diary offers a comprehensive evaluation of symptoms commonly experienced by patients with influenza that can be completed in ≤5 minutes. While other measures of influenza symptom severity exist, such as the Flu-iiQ [7] and the Canadian Acute Respiratory Illness and Flu Scale (CARIFS) [8], these measures do not assess the full range of symptoms associated with varied strains of influenza and identified as important to the patients themselves [9]. Additionally, the CARIFS was developed for use in children, while the FLU-PRO was developed for studies of adults and children, with content validity shown in both groups [9]. Previous research demonstrated that the FLU-PRO is reliable, valid, and responsive to change in hospitalized and non-hospitalized adults with laboratory-confirmed influenza [10]. The purpose of these analyses was to test the reliability, validity, and responsiveness of FLU-PRO scores in patients with influenza symptoms and testing negative for the influenza virus.

## Materials and methods

### Study design

Data from the prospective, observational FLU-PRO development study conducted with informed consent, under the National Institute of Allergy and Infectious Diseases institutional review board approval, and in accordance with the Declaration of Helsinki, were used in these analyses. Methods and results of the primary analyses in those with laboratory-confirmed influenza are reported elsewhere [10].

Briefly, adults ≥18 years of age seeking medical care for acute influenza symptoms at participating clinics in the US (16 sites; English-speaking), Argentina (two sites; Spanish-speaking), United Kingdom (one site; English-speaking), and Mexico (three sites; Spanish-speaking) were recruited during clinic visits. An elevated body temperature of 100°F [37.8°C] or greater was not an enrollment requirement. All study participants were tested for influenza using rapid influenza diagnostic tests (RIDTs), including polymerase chain reaction, rapid antigen test, and/or viral culture by nasal or nasopharyngeal swab. The performance of the FLU-PRO in those testing positive are presented elsewhere [10]. Data from subjects testing negative for the influenza virus were included in the current analysis.

In addition to the influenza diagnostic test, consented patients completed assessments of sociodemographic and clinical characteristics at baseline and a daily diary for 14 days that included the 37-item draft FLU-PRO symptom diary and nine additional questions used for FLU-PRO validation. At the Mexico site, the diary was completed via telephone interview with data entered directly into a web-based portal by the interviewer. Patients in 16 US sites, one UK site, and one Argentina site completed the survey either via interviewer-administration or self-administration via a web-based system using the subject’s personal web-enabled device.

### Compliance with ethical standards

The studies were conducted in accordance with the Declaration of Helsinki, and the National Institute of Allergy and Infectious Diseases ethics committee/institutional review board requirements, and good clinical practice guidelines. Informed consent was obtained from all individual participants included in the study.

### Instruments: Patient-reported Outcomes (PROs)

#### InFLUenza Patient-Reported Outcome (FLU-PRO)

The final FLU-PRO questionnaire instructed respondents to rate the severity of 32 influenza symptoms over the past 24 hours. The presence and severity of influenza signs and symptoms are assessed across six body systems affected by influenza: Nose (4 items), Throat (3 items), Eyes (3 items), Chest/Respiratory (7 items), Gastrointestinal (4 items), and Body/Systemic (11 items). A total score quantifies symptoms overall. Respondents are asked to rate each sign or symptom on a 5-point ordinal severity scale, with higher scores indicating a more severe sign or symptom. The questionnaire was developed for self-report or interviewer-administration, with slight differences in the instructions applicable for each administration.

Development procedures addressing content validity of the FLU-PRO are described elsewhere [9]. Quantitative item reduction and validation testing were performed on data from a prospective validation study, with data from laboratory-confirmed influenza positive patients (N = 221) serving as the primary analytical sample [10].

#### Patient Global Assessments (PGA)

The Patient Global Rating of Flu Severity is a single item to assess participants’ overall influenza symptom severity. Participants were asked to rate severity on the following scale: 0 (“No flu symptoms today”), 1 (“Mild”), 2 (“Moderate”), 3 (“Severe”), and 4 (“Very severe”). The Patient Global Assessment of Interference in Daily Activities is a single item to assess interference in daily activities due to influenza symptoms during that day. Participants rated interference on the following scale: 1 (“Not at all”), 2 (“A little bit”), 3 (“Somewhat”), 4 (“Quite a bit”), and 5 (“Very much”). The Patient Global Assessment of Health is a single item to assess general physical health during that day. Participants rated their physical health on the following scale: 1 (“Poor”), 2 (“Fair”), 3 (“Good”), 4 (“Very good”), and 5 (“Excellent”). Finally, a Patient Global Rating of Change in Flu Severity was used to identify stable patients for reproducibility assessment.

#### Return to “usual” health and activities

Patients were asked to respond (yes/no) to the following questions: “Have you returned to your usual health today?” and “Have you returned to your usual activities today?”

### Statistical analyses

Statistical tests were performed in accordance with classical test theory [11] to evaluate the psychometric properties of the FLU-PRO total and domain scores in participants with ILI, including reliability, construct and known-groups validity, and responsiveness. These analyses were performed on the entire ILI cohort and stratified by hospitalization status.

#### Reliability (internal and test-retest)

Cronbach’s coefficient alpha was used to estimate internal consistency reliability of the FLU-PRO Total and domain scores on day 1. Coefficients of 0.7–0.9 were pre-specified as “good” internal consistency, 0.4–<0.7 as moderate, and <0.4 as low or poor [11,12].

Data from patients whose influenza severity was unchanged over time were used to estimate the test-retest reliability of FLU-PRO Total and domain scores. Stable subjects were defined as those with “no change” on the Patient Global Rating of Change in Flu Severity over two consecutive days during Week 1 (i.e., day 1 to day 2, day 2 to day 3, etc.). If a subject was missing FLU-PRO scores for one of the days in the planned comparison, data for this subject was excluded from that analytical pair. Intraclass correlation coefficients (ICC from a fixed-effects model), paired *t*-tests, and effect size (ES) were calculated to evaluate score reliability. ICCs were expected to be at least moderate, exceeding 0.60. Mean differences between the two observations were expected to be minimal with a small ES (<0.20).

#### Construct validity

The relationship between the FLU-PRO Total and domain scores and three global ratings were assessed using Spearman correlations (*r*_*s*_) using day 1 data, hypothesizing that the relationship between FLU-PRO scores and these global ratings would be moderate to high (*r*_*s*_ >0.30) [13]. Correlations with the Patient Global Rating of Flu Severity were hypothesized to be strongest, while weaker correlations were expected with the more distal constructs, including the Patient Global Rating of Physical Health and the Patient Global Assessment of Interference with Daily Activities.

#### Known-groups validity

Analysis of variance (ANOVA) was used to compare FLU-PRO Total and domain scores across three Patient Global Rating of Flu Severity categories at day 1: “None” or “Mild”; “Moderate”; and “Severe” or “Very severe”. Mean (SD), *F*-scores, and p-values were reported to determine the magnitude of the differences. Pairwise comparisons between means were performed using Scheffe’s test adjusting for multiple comparisons.

#### Responsiveness

Analysis of covariance (ANCOVA) was used to compare changes in FLU-PRO scores at day 7 in responders (those returning to usual health or activity) and non-responders (those not returning to usual health or activity), adjusting for day 1 scores. Responders were defined using the two different anchors in two separate analyses. It was expected that responders would have significantly larger (p <0.05) change scores than non-responders.

#### Exploratory analyses

Exploratory analyses were conducted to statistically compare the symptom profiles of ILI patients in the current analytic sample at day 1 to the influenza positive patients in the FLU-PRO development sample. Specifically, independent samples *t-*tests were used to compare mean FLU-PRO total and domain scores between all ILI patients overall (i.e., hospitalized, and non-hospitalized) versus all influenza positive patients overall, while a 2-way analysis of variance (ANOVA) was used to compare mean FLU-PRO scores by influenza (i.e., positive, and negative) and hospitalization (i.e., hospitalized, and non-hospitalized) status. Scheffe’s test was used to assess pairwise comparisons between groups.

## Results

### Sample

Of the 536 subjects enrolled in the study, 441 had the minimum required data (day 1 diary assessment and ≥1 post-day 1 diary entry); data from the 220 subjects testing negative for the influenza virus were used in the current study. Table 1 presents baseline demographic and clinical characteristics for the analytical sample. The majority of the participants (70.9%) were from non-US countries.

**Table 1:** Patient demographic characteristics by region in patients with influenza like illness (N = 220).

Variable	Day 1	
	USA (n = 64)	Other Countries^1^ (n = 156)
**Age, Years**		
Mean (SD)	32.7 (13.0)	42.1 (15.1)
Median (Min-Max)	27.5 (19–67)	39.0 (20–91)
>65	1 (1.6%)	12 (7.7%)
**Sex, n (%)**		
Female	41 (64.1%)	100 (64.1%)
**Ethnicity, n (%)**		
Hispanic or Latino	5 (7.8%)	152 (97.4%)
Not Hispanic or Latino	59 (92.2%)	4 (2.6%)
**Race, n (%)**		
Asian	2 (3.1%)	0 (0%)
Black or African American	21 (32.8%)	0 (0%)
White or Caucasian (Mestizo)	0 (0%)	152 (97.4%)
White	39 (60.9%)	4 (2.6%)
Other	2 (3.1%)	0 (0%)
**Employment Status, n (%)**		
Employed, full time	19 (29.7%)	81 (51.9%)
Employed, part-time	0 (0%)	20 (12.8%)
Homemaker	1 (1.6%)	23 (14.7%)
Student	0 (0%)	13 (8.3%)
Unemployed	0 (0%)	11 (7.1%)
Retired	0 (0%)	1 (0.6%)
Disabled	0 (0%)	3 (1.9%)
Other	0 (0%)	2 (1.3%)
Missing	44 (68.8%)	2 (1.3%)
**Military Status, n (%)**		
Never in the military	0 (0%)	153 (98.1%)
Active	16 (25.0%)	0 (0%)
Retired	1 (1.6%)	0 (0%)
Reserves	0 (0%)	1 (0.6%)
Other	1 (1.6%)	0 (0%)
Missing	46 (71.9%)	2 (1.3%)
**Highest Level of Education**		
Elementary/primary school	0 (0%)	15 (9.6%)
Secondary/high school	5 (7.8%)	54 (34.6%)
Some college	7 (10.9%)	8 (5.1%)
College degree	4 (6.3%)	55 (35.3%)
Postgraduate degree	5 (7.8%)	13 (8.3%)
Other	43 (67.2%)	11 (7.1%)
**Current Treatments, n (%)**		
Oseltamivir (Tamiflu)	4 (6.3%)	17 (10.9%)
Amantadine (Symmetrel)	0 (0%)	6 (3.8%)
Other	15 (23.4%)	93 (59.6%)
None	45 (70.3%)	52 (33.3%)
**Co-morbidities, n (%)**		
None	44 (68.8%)	67 (42.9%)
Asthma	8 (12.5%)	14 (9.0%)
Chronic Obstructive Pulmonary Disease (COPD)	0 (0%)	3 (1.9%)
Osteoporosis	0 (0%)	4 (2.6%)
Depression	1 (1.6%)	14 (9.0%)
Hypertension	2 (3.1%)	28 (17.9%)
Raised cholesterol	1 (1.6%)	18 (11.5%)
Stomach ulcers	0 (0%)	5 (3.2%)
Heart attack/angina	0 (0%)	5 (3.2%)
Diabetes	3 (4.7%)	20 (12.8%)
Kidney disease	2 (3.1%)	10 (6.4%)
Lung disease	0 (0%)	8 (5.1%)
Tuberculosis	0 (0%)	3 (1.9%)
Other	6 (9.4%)	46 (29.5%)

^1^Other countries include Mexico (n = 154), Argentina (n = 1), and UK (n = 1).

### Evaluation of psychometric properties

Results for the entire sample are reported below; results stratified by hospitalization status are provided in the online supplement S1 File.

#### Descriptive statistics of FLU-PRO domain and Total scores

The distributional characteristics of the FLU-PRO domain and Total scores on day 1 are shown in Table 2. Mean domain scores were lowest for the Gastrointestinal domain (mean = 0.5; SD = 0.7) and highest for the Nose (mean = 1.5; SD = 1.0) and Throat (mean = 1.5; SD = 1.2) domains. Floor effects were found for the Gastrointestinal (47%) domain. No ceiling effects were found.

**Table 2:** FLU-PRO domain and total score descriptive statistics Day 1.

	N	Mean ± SD	Range, Median (Mode)	Floor n (%)	Ceiling n (%)	Missing n (%)
Nose	218^1^	1.5 ± 1.0	0.0–3.8, 1.5 (0.3)	20 (9.2%)	0 (0.0%)	2 (0.9%)
Throat	218^1^	1.5 ± 1.2	0.0–4.0, 1.3 (0.3)	26 (11.9%)	5 (2.3%)	2 (0.9%)
Eyes	218^1^	0.9 ± 1.0	0.0–4.0, 0.7 (0.0)	61 (28.0%)	3 (1.4%)	2 (0.9%)
Chest/ Respiratory	218^1^	1.4 ± 0.8	0.0–3.7, 1.3 (1.3)	9 (4.1%)	0 (0.0%)	2 (0.9%)
Gastrointestinal	219	0.5 ± 0.7	0.0–3.5, 0.3 (0.0)	103 (47.0%)	0 (0.0%)	1 (0.5%)
Body/Systemic	219	1.4 ± 0.9	0.0–3.6, 1.3 (0.7)	2 (0.9%)	0 (0.0%)	1 (0.5%)
Total Score	218^1^	1.3 ± 0.6	0.0–3.1, 1.2 (0.7)	0 (0.0%)	0 (0.0%)	2 (0.9%)

Note: higher FLU-PRO scores = more severe symptoms.

^1^ One item was found missing.

Abbreviations: SD = Standard Deviation

#### Reliability (internal and test-retest)

Internal consistency reliability (Cronbach’s alpha) was high for all domains (Nose = 0.80, Throat = 0.84, Eyes = 0.79, Chest/Respiratory = 0.75, Gastrointestinal = 0.72; Body/Systemic = 0.86) and the Total score (0.90).

Two-day test-retest reliability during Week 1 are shown in Table 3. Across all two-day assessment periods, FLU-PRO Score ICC and ES estimates were strong. Score reproducibility early in the evaluation (between days 1 and 2) was lowest (Total: 0.64), while reproducibility in the latter days (2–3, 4–5, 5–6) were higher (Total: 0.90, 0.89, 0.88, respectively).

**Table 3:** Two-day test-retest reliability of FLU-PRO scores during Week 1 (Days 1 to Day 7).

FLU-PRO Scores	N^1^	Day X Mean (SD)	Day Y Mean (SD)	Mean Difference (SD)^2^	T Statistic	p value	Effect Size	ICC
**Day 1 to Day 2**								
Nose	50	1.8 (1.0)	1.7 (0.9)	0.1 (0.6)	1.05	0.3010	0.09	0.78
Throat	50	1.7 (1.2)	1.4 (1.1)	0.3 (0.9)	2.26	0.0283	0.26	0.65
Eyes	50	1.0 (1.0)	1.0 (0.9)	0.1 (0.8)	0.52	0.6041	0.06	0.65
Chest/Respiratory	50	1.4 (0.8)	1.3 (0.7)	0.0 (0.6)	0.51	0.6142	0.05	0.73
Gastrointestinal	50	0.4 (0.7)	0.3 (0.5)	0.1 (0.6)	1.31	0.1965	0.16	0.46
Body/Systemic	50	1.4 (0.7)	1.2 (0.7)	0.3 (0.7)	2.68	0.0099	0.37	0.50
Total Score	50	1.3 (0.5)	1.2 (0.5)	0.2 (0.4)	2.56	0.0136	0.29	0.64
**Day 2 to Day 3**								
Nose	44	1.4 (0.8)	1.4 (0.8)	0.1 (0.4)	0.84	0.4042	0.07	0.86
Throat	44	1.4 (1.2)	1.2 (0.9)	0.2 (0.8)	1.58	0.1210	0.15	0.74
Eyes	44	0.8 (0.8)	0.7 (0.9)	0.1 (0.7)	0.84	0.4037	0.11	0.70
Chest/Respiratory	44	1.4 (0.8)	1.5 (0.8)	-0.1 (0.5)	-1.25	0.2191	0.13	0.78
Gastrointestinal	45	0.4 (0.7)	0.4 (0.5)	-0.0 (0.6)	-0.39	0.6958	0.05	0.55
Body/Systemic	44	1.1 (0.8)	1.0 (0.7)	0.1 (0.4)	1.97	0.0550	0.16	0.83
Total Score	44	1.1 (0.6)	1.1 (0.6)	0.1 (0.3)	1.34	0.1859	0.08	0.90
**Day 3 to Day 4**								
Nose	41	1.3 (0.9)	1.3 (0.9)	0.0 (0.5)	0.00	1.0000	0.00	0.82
Throat	41	1.3 (1.1)	1.0 (1.0)	0.3 (0.6)	2.52	0.0158	0.23	0.79
Eyes	41	0.7 (0.9)	0.5 (0.7)	0.2 (0.7)	1.47	0.1485	0.19	0.56
Chest/Respiratory	41	1.4 (0.8)	1.4 (0.7)	0.0 (0.5)	0.29	0.7727	0.03	0.81
Gastrointestinal	42	0.2 (0.3)	0.2 (0.4)	0.0 (0.4)	0.38	0.7026	0.07	0.46
Body/Systemic	42	0.9 (0.8)	0.8 (0.5)	0.1 (0.6)	0.79	0.4361	0.09	0.63
Total Score	41	1.0 (0.6)	0.9 (0.5)	0.1 (0.4)	1.19	0.2407	0.12	0.75
**Day 4 to Day 5**								
Nose	30	0.9 (0.8)	0.8 (0.6)	0.1 (0.4)	1.27	0.2156	0.11	0.86
Throat	30	0.7 (0.9)	0.8 (0.9)	-0.1 (0.7)	-0.44	0.6649	0.06	0.72
Eyes	30	0.5 (0.8)	0.3 (0.6)	0.2 (0.5)	2.34	0.0264	0.25	0.76
Chest/Respiratory	30	1.1 (0.6)	1.0 (0.7)	0.1 (0.3)	1.28	0.2092	0.13	0.86
Gastrointestinal	31	0.2 (0.4)	0.3 (0.5)	-0.1 (0.3)	-0.94	0.3536	0.15	0.69
Body/Systemic	31	0.7 (0.6)	0.7 (0.7)	-0.0 (0.4)	-0.71	0.4824	0.08	0.83
Total Score	30	0.7 (0.5)	0.7 (0.5)	0.0 (0.2)	0.42	0.6787	0.04	0.89
**Day 5 to Day 6**								
Nose	32	0.8 (0.6)	0.8 (0.6)	-0.0 (0.4)	-0.22	0.8249	0.03	0.76
Throat	32	0.7 (1.1)	0.7 (1.0)	0.0 (0.2)	1.07	0.2922	0.04	0.98
Eyes	32	0.4 (0.7)	0.4 (0.6)	0.0 (0.5)	0.24	0.8154	0.03	0.71
Chest/Respiratory	32	1.0 (0.7)	1.0 (0.8)	0.0 (0.5)	0.10	0.9182	0.01	0.79
Gastrointestinal	32	0.3 (0.4)	0.2 (0.4)	0.0 (0.3)	0.67	0.5089	0.09	0.70
Body/Systemic	32	0.7 (0.6)	0.6 (0.6)	0.1 (0.3)	1.99	0.0554	0.17	0.87
Total Score	32	0.7 (0.5)	0.7 (0.5)	0.0 (0.2)	1.09	0.2861	0.09	0.88
**Day 6 to Day 7**								
Nose	27	0.8 (0.7)	0.9 (0.6)	-0.1 (0.4)	-0.74	0.4637	0.08	0.83
Throat	27	0.7 (0.9)	0.8 (0.9)	-0.1 (0.5)	-0.66	0.5181	0.07	0.86
Eyes	27	0.2 (0.3)	0.2 (0.3)	-0.0 (0.2)	-0.27	0.7873	0.04	0.64
Chest/Respiratory	27	1.0 (0.7)	1.1 (0.6)	-0.1 (0.5)	-0.88	0.3885	0.12	0.70
Gastrointestinal	27	0.1 (0.3)	0.1 (0.2)	-0.0 (0.2)	-0.63	0.5372	0.07	0.82
Body/Systemic	27	0.5 (0.5)	0.3 (0.3)	0.1 (0.3)	2.26	0.0325	0.27	0.73
Total Score	27	0.6 (0.4)	0.6 (0.3)	0.0 (0.2)	0.24	0.8106	0.02	0.85

^1^Number of study participants with no change in flu symptom at day Y. Stable subjects were defined as those with “no change” on the Patient Global Rating of Change in Flu Severity using two consecutive days.

^2^Mean difference = average Day X FLU-PRO score average Day Y FLU-PRO score (ex. Day 1 score Day 2 score); p value from paired t-test.

#### Construct validity

As shown in Table 4, at day 1 the strongest associations were observed between FLU-PRO domain and Total scores and the Patient Global Rating of Flu Severity (r_s_ = 0.31–0.68, p<0.0001), except Gastrointestinal (r_s_ = 0.19, p<0.05). Moderate to high correlations were also found between FLU-PRO scores and the Patient Global Rating of Physical Health for all scores (r_s_ = -0.30–-0.49) except Gastrointestinal (r_s_ = -0.21), Nose, and Eyes (both r_s_ = -0.28). Only associations between the Patient Global Assessment of Interference in Daily Activities and the Total score (r_s_ = 0.36, p<0.0001) and Body/Systemic domain score (r_s_ = 0.40, p<0.0001) were moderate to strong.

**Table 4:** Construct validity: FLU-PRO scale correlations with other PRO measures at Day 1.

	Domains and Total Score, r (p-value)^1^						
Day 1	Nose	Throat	Eyes	Chest/ Respiratory	Gastrointestinal	Body/ Systemic	Total Score
Patient Global Rating of Flu Severity^**2**^	0.44***	0.47***	0.48***	0.31***	0.19*	0.63***	0.68***
Patient Global Rating of Physical Health^**3**^	-0.28***	-0.31***	-0.28***	-0.30***	-0.21*	-0.43***	-0.49***
Patient Global Assessment of Interference in Daily Activities^**4**^	0.07	0.11	0.17*	0.27***	0.19*	0.40***	0.36***

^1^Spearman correlation coefficients: ***p<0.0001, *p<0.05

^2^Greater values indicate greater disease severity

^3^Greater values indicate better patient health

^4^Greater values indicate greater interference with daily activities

#### Known-groups validity

Significant differences in FLU-PRO total scores were found across patient groups according to the patient global symptom severity rating (F = 81.7, p<0.001). Mean [SD] scores were lowest in the No/Mild Symptoms group (0.81 [0.45]), followed by the Moderate (1.28 [0.48]) and Severe/Very Severe groups (1.84 [0.49]). All pairwise comparisons using Scheffe’s test were statistically significant (p<0.001). A similar pattern of mean values was demonstrated for all FLU-PRO domain scores. Further, pairwise comparisons for each domain score showed a similar pattern to the Total score, with the exception of the No/Mild symptoms versus Moderate for the Chest/Respiratory and Gastrointestinal domains, and Moderate versus Severe/Very Severe for the Nose domain, which were in the correct direction but nonsignificant (p>0.05) (Table 5).

**Table 5:** Known-groups validity: FLU-PRO scores by patient global rating of disease severity, Day 1.

	Patient Global Rating of Flu Severity						
	No/Mild		Severe and				
	Symptoms (N = 81)	Moderate (N = 77)	Very Severe (N = 60)				
Scale	Mean (SD)	Mean (SD)	Mean (SD)	F Value (p-value)^1^	Pairwise Comparisons: No/Mild vs Moderate (p-value)	Pairwise Comparisons: No/Mild vs Severe/Very Severe (p-value)	Pairwise Comparisons: Moderate vs Severe/Very Severe (p-value)
Nose	0.89 (0.74)	1.65 (0.98)	2.00 (1.09)	26.9***	p<0.001	p<0.001	NS
Throat	0.97 (0.98)	1.48 (0.99)	2.41 (1.21)	32.4***	p<0.05	p<0.001	p<0.001
Eyes	0.37 (0.52)	1.04 (0.84)	1.48 (1.18)	30.7***	p<0.001	p<0.001	p<0.05
Chest/Respiratory	1.14 (0.73)	1.42 (0.69)	1.79 (0.93)	12.0***	NS	p<0.001	p<0.05
Gastrointestinal	0.32 (0.61)	0.38 (0.53)	0.72 (0.89)	6.9**	NS	p<0.01	p<0.05
Body/Systemic	0.83 (0.65)	1.40 (0.71)	2.15 (0.75)	62.2***	p<0.001	p<0.001	p<0.001
Total Score	0.81 (0.45)	1.28 (0.48)	1.84 (0.49)	81.7***	p<0.001	p<0.001	p<0.001

^1^p values are: **<0.01, ***<0.001.

Abbreviations: SD = Standard Deviation; NS = Non-significant

#### Responsiveness

**Fig 1:**
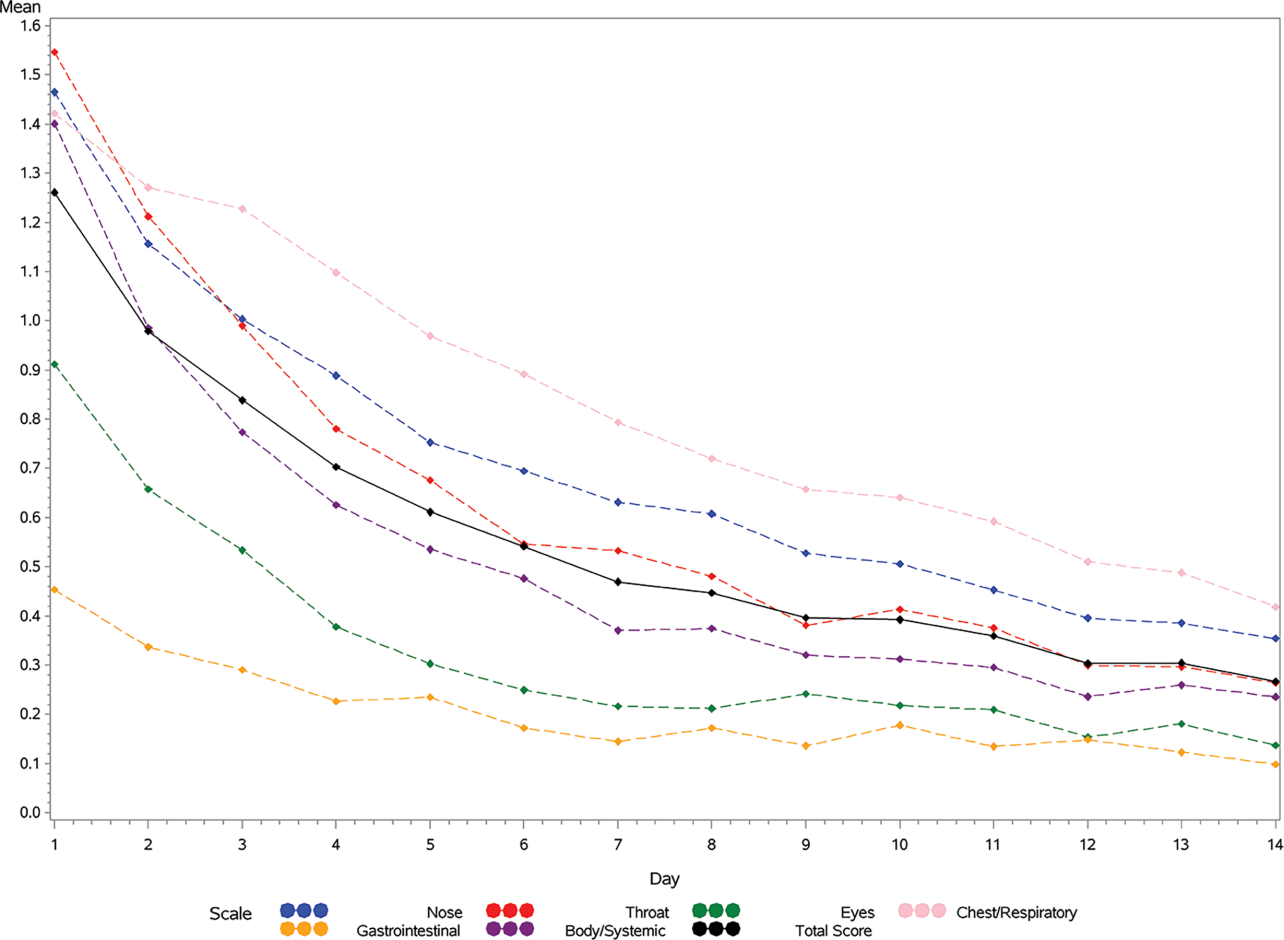
FLU-PRO domain and total score by diary days 1 to 14: Influenza-Negative Patients.

Change in FLU-PRO total and domain scores across time are shown in Fig 1, demonstrating a reduction in mean scores across days 1–14. In support of responsiveness, mean total and domain change scores were significantly greater for patients reporting a return to usual health (responders) by day 7, compared to those who did not, with the exception of the Eyes and Gastrointestinal domains (Table 6). Using patient’s report of return to usual activities as an anchor for responsiveness, mean Total and Body/Systemic change scores were significantly greater for responders, while all other comparisons failed to reach statistical significance (p>0.05).

**Table 6:** Responsiveness of FLU-PRO by patient return to usual health (N = 169)^1^ and usual activities (N = 117)^2^, Day 1 to Day 7.

	Responders^3^			Non-Responders			
Scale	Day 1 Mean (SD)	Day 7 Mean (SD)	Change Score LSMean (SDerr)	Day 1 Mean (SD)	Day 7 Mean (SD)	Change Score LSMean (SDerr)	p-value^4^
**Nose**							
Usual Health	1.5 (1.0)	0.5 (0.5)	1.0 (0.1)	1.4 (1.0)	0.8 (0.7)	0.7 (0.1)	0.0003
Usual Activities	1.6 (1.1)	0.7 (0.7)	0.7 (0.1)	0.9 (0.8)	0.5 (0.6)	0.7 (0.1)	0.8751
**Throat**							
Usual Health	1.5 (1.1)	0.3 (0.5)	1.2 (0.1)	1.7 (1.3)	0.7 (0.8)	0.9 (0.1)	0.0029
Usual Activities	1.8 (1.2)	0.5 (0.7)	1.1 (0.1)	1.1 (1.2)	0.5 (0.9)	0.9 (0.1)	0.1351
**Eyes**							
Usual Health	0.9 (1.1)	0.1 (0.4)	0.7 (0.1)	0.9 (0.9)	0.3 (0.6)	0.6 (0.0)	0.1202
Usual Activities	1.0 (1.1)	0.2 (0.5)	0.7 (0.1)	0.6 (0.9)	0.2 (0.6)	0.6 (0.1)	0.0919
**Chest/Respiratory**							
Usual Health	1.3 (0.7)	0.5 (0.6)	0.9 (0.1)	1.5 (0.8)	1.0 (0.7)	0.5 (0.1)	< .0001
Usual Activities	1.4 (0.8)	0.8 (0.7)	0.7 (0.1)	1.6 (0.8)	0.8 (0.8)	0.7 (0.1)	0.6139
**Gastrointestinal**							
Usual Health	0.4 (0.7)	0.1 (0.3)	0.3 (0.0)	0.4 (0.6)	0.2 (0.4)	0.2 (0.0)	0.0585
Usual Activities	0.4 (0.7)	0.1 (0.2)	0.4 (0.0)	0.4 (0.6)	0.2 (0.5)	0.3 (0.0)	0.0777
**Body/Systemic**							
Usual Health	1.3 (0.9)	0.2 (0.3)	1.2 (0.1)	1.4 (0.9)	0.5 (0.5)	0.9 (0.0)	< .0001
Usual Activities	1.6 (0.9)	0.3 (0.4)	1.2 (0.0)	1.1 (0.9)	0.5 (0.5)	0.9 (0.1)	0.0004
**Total Score**							
Usual Health	1.2 (0.6)	0.3 (0.3)	0.9 (0.0)	1.3 (0.6)	0.6 (0.5)	0.7 (0.0)	< .0001
Usual Activities	1.4 (0.6)	0.5 (0.4)	0.9 (0.0)	1.1 (0.6)	0.5 (0.5)	0.7 (0.1)	0.0064

^1^Responders: N = 64; Non-responders: N = 105

^2^Responders: N = 69; Non-responders: N = 48

^3^Responders are defined as patients responding that they have returned to their usual health or usual activities at Day 7.

^4^Analysis of covariance was used to compare changes in FLU-PRO scores at day 7 in responders and non-responders, adjusting for day 1 scores.

### Exploratory comparisons between influenza positive and ILI patient scores

Patients with confirmed influenza had statistically significantly greater symptom severity at day 1 compared to patients with ILI, including FLU-PRO Total (p<0.0001), Nose (p<0.05), Chest/Respiratory (p<0.0001), Gastrointestinal (p<0.01), and Body/Systemic scores (p<0.0001) (Table L and Figure C in S1 File). The Throat and Eyes domains were not significantly different between groups (p>0.05). Scores were also examined by hospitalization status. In those not hospitalized, ILI patients reported significantly greater Throat scores than the influenza positive patients (p = 0.0174); while the influenza positive patients reported significantly greater Chest/Respiratory, Body/Systemic, and Total scores compared to ILI patients (p<0.05) (Figure D in S1 File). Among hospitalized patients, those with confirmed influenza reported significantly greater Nose, Gastrointestinal, Body/Systemic, and Total score compared to ILI patients (all p<0.05) (Figure E in S1 File). In addition, for both patients with confirmed influenza and ILI, the Patient Global Rating of Flu Severity scores decreased from day 1 to 14, as was seen with FLU-PRO total and domain scores (Figure F in S1 File).

## Discussion

The objective of this study was to assess the performance properties of the FLU-PRO in patients seen in the clinic with ILI and testing negative for the influenza virus. For FDA qualification purposes, the FLU-PRO was developed and tested in patients with acute, laboratory-confirmed influenza, with scores exhibiting sound measurement properties in the sample overall and stratified by hospitalization status. This current study tested the properties of FLU-PRO scores in hospitalized and non-hospitalized patients with ILI. If the instrument performs well, this would facilitate its use in population-level epidemiologic studies and natural history studies, where laboratory diagnosis of influenza is not always sought or confirmed, and in studies of patients with influenza-like symptoms but infected with viruses or pathogens other than influenza.

Results of this study indicate FLU-PRO scores are reliable and reproducible, demonstrate construct and known-groups validity, and are responsive to change in subjects with ILI. Internal consistency levels were high for each of the domains and the total score, and 2-day test-retest reliability levels during the first seven days following enrollment were generally moderate to strong. The relatively low test-retest reliability of the Gastrointestinal domain is due, in part, to the low symptom prevalence and constrained variance. Further evaluation in patients with influenza strains or pathogens characterized by greater incidence of gastrointestinal distress should be performed, as gastrointestinal symptoms may occur in up to 40% of patients with influenza [14]. It was interesting to note that score reproducibility was lower during the first 2 assessment days in “stable” patients (those reporting “no change” in their symptoms). This may be a reflection of the relative nature of “no change” during recovery from an acute illness, an interpretation that could be explored qualitatively.

As hypothesized and in support of construct validity, FLU-PRO scores were significantly related to patient global ratings of influenza severity and global health, with patterns and values similar to those found in the influenza positive sample. Weaker associations were observed with the patient global rating of interference with activities, as patients may return to usual activities before total improvement/resolution of symptoms. The data similarly supported known-groups validity as FLU-PRO scores were lowest in patients rating their symptoms as the mildest, and increased with increasing patient-reported symptom severity. It was interesting to note that eye, nose, and throat symptoms were more strongly related to flu severity than respiratory symptoms in milder cases (defined by patient global assessment), with respiratory symptoms sensitive to differences in the more severe cases. These results suggest the non-respiratory domains may be particularly useful in studies of patients with milder influenza-like illness and to capture the full range of symptom severity. The low prevalence of gastrointestinal symptoms (relatively high percentage of participants reporting no gastrointestinal symptoms) would make it more difficult to show significant differences by patient global ratings of influenza severity. Further study in patients with strains or pathogens characterized by gastrointestinal effects are needed.

When responders were defined by reports of return to usual health, the FLU-PRO demonstrated responsiveness to change from day 1–7. Similar to results observed in the influenza positive sample, FLU-PRO total and domain scores, except for Eyes and Gastrointestinal, were responsive to change in the ILI group when return to health was used to define response. Using return to usual activities as an anchor, responsiveness in ILI patients was shown in the total and Body/Systemic domain scores.

Comparing current study findings in patients with ILI to influenza-positive patients in the original validation study sample [10], the FLU-PRO performed similarly in both patient groups. FLU-PRO domain and total score reliability and reproducibility was supported, with similar coefficient profiles across domains. Additionally, there were moderate to strong correlations between FLU-PRO domain and total scores and Patient Global Rating of Flu Severity, Correlations with interference with daily activities were stronger in the laboratory-confirmed influenza patient population.

Results of this study show the utility of the multi-domain profile scores provided by the FLU-PRO. Unlike other measures that assess 1 or 2 dimensions of influenza symptom severity for use in either adults or children [7,8], the FLU-PRO is a comprehensive, multi-dimensional measure with content intended for use across a wide age spectrum [9]. Its structure yields a total score, representing symptom severity overall to facilitate hypothesis testing, and separate assessments of 6 different bodily systems that may be differentially affected by the virus and/or treatment. For example, the FLUiiQ assesses two domains: respiratory and systemic domains, with cough, sore throat, and nasal congestion symptoms captured within a single domain [7]. However, the ILI experienced by the patients in the current study was characterized by sore throat, with less severe respiratory and systemic symptoms than observed in patients testing positive for the influenza virus. The detailed body system profile can capture these symptoms which patients stated were important to them [9], and also provide information on where treatment is and is not providing symptomatic relief.

Gastrointestinal symptoms were experienced by less than 25% of the sample, indicating these symptoms were not prevalent in the year of this study but have been observed in a greater proportion of patients in other years with different circulating viral strains. Although this is technically a floor effect, the results provided quantitative information on the prevalence of gastrointestinal symptoms in this sample [14]. The Gastrointestinal domain should be retained, to assure comprehensive symptom assessment across viral strains or pathogens.

While the findings of the current study suggest FLU-PRO scores are reliable, valid, and responsive in this expanded target population, the study had several limitations. First, although patients were tested for influenza using RIDTs, the sensitivity and sensitivity of these tests for detecting influenza A or B can vary, and can differ pending the strain of influenza circulating during any given year [15,16]. Thus, it is likely that some ILI patients in this study were infected with influenza A or B. Testing for viruses other than influenza was not available at most sites, precluding analyses by viral etiology. The FLU-PRO performance can be evaluated in various specific viral infections in future studies. It is important to note that the purpose of the FLU-PRO is not to diagnose or to differentiate influenza types. Rather, it is to quantify the presence and severity of symptoms that are characteristic of various influenza and other respiratory viruses. The FLU-PRO performed well in those testing positive for influenza [10], the primary analyses of the study, with the current study suggesting the performance properties were comparable in patients presenting to the clinic with a similar set of symptoms but testing negative for influenza using the RIDT. Second, although hospitalized patients were included in the analytic sample, details about the hospitalization event (e.g., duration of influenza prior to hospitalization, acuity level during hospitalization, duration of hospitalization, complicating comorbid conditions, treatment) are unknown. These can be evaluated in future studies, but seem unlikely to affect the measurement properties of the instrument, although may result in a different scoring algorithm. Third, due to sample size limitations, stratified analyses by patient characteristics (e.g., sex, age) was not performed, but should be considered for future studies. Finally, while the content validity of the FLU-PRO has been established in children and adolescents through qualitative research, quantitative evaluation of measurement properties has not been conducted in these patient groups.

Results of this study suggest the FLU-PRO may be useful as an outcome measure in clinical trials and epidemiological studies of disease due to non-influenza viruses. Although influenza is often indistinguishable from other viral infections in clinical practice, a standardized comprehensive symptom measure may be useful for exploring differences in the presentation and course of various viral infections. The FLU-PRO may also be used as an inclusion criteria for studies to ensure patients have sufficient symptoms to test treatment effects and recovery patterns. Future work also may evaluate the use of the FLU-PRO in prevention studies, such as vaccines, for disease due to various viruses including influenza.

## Conclusion

Results of this study suggest FLU-PRO scores are reliable, valid, and responsive to change in patients testing negative for the influenza virus, indicating the instrument can be used in studies of confirmed influenza and influenza-like illness.

## Supporting information

S1 File **Online supplement: Table A.** Patient Demographic and Clinical Characteristics by Hospitalization Status (N = 220) **Table B.** FLU-PRO Domain and Total Score Descriptive Statistics in Non-Hospitalized Patients—Day 1 **Table C.** FLU-PRO Domain and Total Score Descriptive Statistics in Hospitalized Patients—Day 1 **Table D.** Two-day Reliability of FLU-PRO—Days 1 to Day 7 in Non-Hospitalized Patients **Table E.** Two-day Reliability of FLU-PRO—Days 1 to Day 7 in Hospitalized Patients **Table F.** Construct Validity: FLU-PRO Scale Correlations with Other PRO Measures at Day 1 in Non-Hospitalized Patients **Table G.** Construct Validity: FLU-PRO Scale Correlations with Other PRO Measures at Day 1 in Hospitalized Patients **Table H.** Known-Groups Validity: FLU-PRO Scores by Patient Global Rating of Disease Severity, Day 1 in Non-Hospitalized Patients **Table I.** Known-Groups Validity: FLU-PRO Scores by Patient Global Rating of Disease Severity, Day 1 in Hospitalized Patients **Table J.** Responsiveness of FLU-PRO by Patient Return to Usual Health (N = 119)^1^ and Usual Activities (N = 67)^2^, Day 1 to Day 7 in Non-Hospitalized Patients **Table K.** Responsiveness of FLU-PRO by Patient Return to Usual Health (N = 50)^1^ and Usual Activities (N = 50)^2^, Day 1 to Day 7 in Hospitalized Patients **Table L.** FLU-PRO Domain and Total Score Descriptive Statistics by Influenza Status—Day 1 **Table M.** 2-Way ANOVA of FLU-PRO Domain and Total Score by Influenza and Hospitalization Status—Day 1 **Figure A.** FLU-PRO Domain and Total Score by Diary Days 1 to 14: Non-Hospitalized Influenza-Negative Patients **Figure B.** FLU-PRO Domain and Total Score by Diary Days 1 to 14: Hospitalized Influenza-Negative Patients **Figure C.** FLU-PRO Domain and Total Score Descriptive Statistics—Day 1 **Figure D.** FLU-PRO Domain and Total Score Descriptive Statistics in Non-Hospitalized Patients—Day 1 **Figure E.** FLU-PRO Domain and Total Score Descriptive Statistics in Hospitalized Patients—Day 1 **Figure F.** Patient Global Rating of Flu Severity- Day 1 to 14.
(DOCX)

## References

[1] Centers for Disease Control and Prevention (CDC) (2016) Key Facts about Influenza (Flu) & Flu Vaccine.

[2] Centers for Disease Control and Prevention (CDC) (2016) Overview of Influenza Surveillance in the United States.

[3] Frost MH, Reeve BB, Liepa AM, Stauffer JW, Hays RD, et al (2007) What is sufficient evidence for the reliability and validity of patient-reported outcome measures? Value in Health 10: S94–S105. doi: 10.1111/j.1524-4733.2007.00272.x 17995479 10.1111/j.1524-4733.2007.00272.x

[4] Revicki DA (2007) FDA draft guidance and health-outcomes research. Lancet 369: 540–542. doi: 10.1016/S0140-6736(07)60250-5 17307086 10.1016/S0140-6736(07)60250-5

[5] Rothman M, Burke L, Erickson P, Leidy NK, Patrick DL, et al (2009) Use of existing patient-reported outcome (PRO) instruments and their modification: the ISPOR Good Research Practices for Evaluating and Documenting Content Validity for the Use of Existing Instruments and Their Modification PRO Task Force Report. Value Health 12: 1075–1083. doi: 10.1111/j.1524-4733.2009.00603.x 19804437 10.1111/j.1524-4733.2009.00603.x

[6] Food Drug Administration (FDA) (2009) Guidance for industry patient-reported outcome measures: use in medical product development to support labeling claims. Fed Regist 74: 65132–65133.

[7] Osborne RH, Norquist JM, Elsworth GR, Busija L, Mehta V, et al (2011) Development and validation of the influenza intensity and impact questionnaire (FluiiQ™). Value in health 14: 687–699. doi: 10.1016/j.jval.2010.12.005 21839407 10.1016/j.jval.2010.12.005

[8] Jacobs B, Young NL, Dick PT, Ipp MM, Dutkowski R, et al (2000) Canadian Acute Respiratory Illness and Flu Scale (CARIFS): development of a valid measure for childhood respiratory infections. J Clin Epidemiol 53: 793–799. 10942861 10.1016/s0895-4356(99)00238-3

[9] Powers JH, Guerrero ML, Leidy NK, Fairchok MP, Rosenberg A, et al (2016a) Development of the Flu-PRO: a patient-reported outcome (PRO) instrument to evaluate symptoms of influenza. BMC Infect Dis 16: 1. 26729246 10.1186/s12879-015-1330-0 PMC4700740

[10] Powers J, Bacci E, Leidy N, Stringer S, Kim K, et al (2016b) Evaluation of the Performance Properties of the Influenza Patient-Reported Outcomes Instrument (Flu-Pro). Value in Health 19: A220–A221.

[11] Nunnally JC, Bernstein IH (1994) Psychometric theory New York: McGraw-Hill xxiv, 752 p. p.

[12] Cronbach LJ (1951) Coefficient alpha and the internal structure of tests. Psychometrika 16: 297–334.

[13] Cohen J (1988) Statistical Power Analysis for the Behavioral Sciences. Hillsdale, NJ: Lawrence Erlbaum Associates Inc,.

[14] Minodier L, Charrel RN, Ceccaldi PE, van der Werf S, Blanchon T, et al (2015) Prevalence of gastrointestinal symptoms in patients with influenza, clinical significance, and pathophysiology of human influenza viruses in faecal samples: what do we know? Virol J 12: 215 doi: 10.1186/s12985-015-0448-4 26651485 10.1186/s12985-015-0448-4 PMC4676820

[15] Dunn JJ, Gordon C, Kelley C, Carroll KC (2003) Comparison of the Denka-Seiken INFLU A.B-Quick and BD Directigen Flu A+B kits with direct fluorescent-antibody staining and shell vial culture methods for rapid detection of influenza viruses. J Clin Microbiol 41: 2180–2183. doi: 10.1128/JCM.41.5.2180-2183.2003 12734274 10.1128/JCM.41.5.2180-2183.2003 PMC154708

[16] Ozdemir M, Yavru S, Baysal B (2012) Comparison of the detection of influenza A and B viruses by different methods. J Int Med Res 40: 2401–2408. doi: 10.1177/030006051204000639 23321198 10.1177/030006051204000639

