# Supplementary material for: Performance of the inFLUenza Patient-Reported Outcome (FLU-PRO) diary in patients with influenza-like illness (ILI)
Source: PLoS One. 2018 Mar 22;13(3):e0194180. doi: 10.1371/journal.pone.0194180 (PMC5863969; doi:10.1371/journal.pone.0194180)
Supplement: S1 File — Online supplement: Table A. Patient Demographic and Clinical Characteristics by Hospitalization Status (N = 220) Table B. FLU-PRO Domain and Total Score Descriptive Statistics in Non-Hospitalized Patients—Day 1 Table C. FLU-PRO Domain and Total Score Descriptive Statistics in Hospitalized Patients—Day 1 Table D. Two-day Reliability of FLU-PRO—Days 1 to Day 7 in Non-Hospitalized Patients Table E. Two-day Reliability of FLU-PRO—Days 1 to Day 7 in Hospitalized Patients Table F. Construct Validity: FLU-PRO Scale Correlations with Other PRO Measures at Day 1 in Non-Hospitalized Patients Table G. Construct Validity: FLU-PRO Scale Correlations with Other PRO Measures at Day 1 in Hospitalized Patients Table H. Known-Groups Validity: FLU-PRO Scores by Patient Global Rating of Disease Severity, Day 1 in Non-Hospitalized Patients Table I. Known-Groups Validity: FLU-PRO Scores by Patient Global Rating of Disease Severity, Day 1 in Hospitalized Patients Table J. Responsiveness of FLU-PRO by Patient Return to Usual Health (N = 119)1 and Usual Activities (N = 67)2, Day 1 to Day 7 in Non-Hospitalized Patients Table K. Responsiveness of FLU-PRO by Patient Return to Usual Health (N = 50)1 and Usual Activities (N = 50)2, Day 1 to Day 7 in Hospitalized Patients Table L. FLU-PRO Domain and Total Score Descriptive Statistics by Influenza Status—Day 1 Table M. 2-Way ANOVA of FLU-PRO Domain and Total Score by Influenza and Hospitalization Status—Day 1 Figure A. FLU-PRO Domain and Total Score by Diary Days 1 to 14: Non-Hospitalized Influenza-Negative Patients Figure B. FLU-PRO Domain and Total Score by Diary Days 1 to 14: Hospitalized Influenza-Negative Patients Figure C. FLU-PRO Domain and Total Score Descriptive Statistics—Day 1 Figure D. FLU-PRO Domain and Total Score Descriptive Statistics in Non-Hospitalized Patients—Day 1 Figure E. FLU-PRO Domain and Total Score Descriptive Statistics in Hospitalized Patients—Day 1 Figure F. Patient Global Rating of Flu Severity- Day 1 to 14. (DOCX) [file pone.0194180.s001.docx]

# ONLINE SUPPLEMENT

# SUPPLEMENTARY TABLES: STRATIFIED ANALYSES BY HOSPITALIZATION STATUS

Evaluation of the Performance Properties of the InFLUenza Patient-Reported Outcome (FLU-PRO^©^) Instrument in Patients with Influenza Like Illness

Table A in S1 File. Patient Demographic and Clinical Characteristics by Hospitalization Status (N=220)

| **Variable** | **Day 1** | |
| --- | --- | --- |
|  | **Hospitalization (n=61)** | **No Hospitalization (n=159)** |
| **Age, Years** |  |  |
| Mean (SD) | 48.9 (16.8) | 35.7 (12.7) |
| Median (Range) | 50.0 (20 - 91) | 34.0 (19 - 72) |
| >65 | 9 (14.8%) | 4 (2.5%) |
| **Sex, n (%)** |  |  |
| Female | 36 (59.0%) | 105 (66.0%) |
| **Ethnicity, n (%)** |  |  |
| Hispanic or Latino | 60 (98.4%) | 97 (61.0%) |
| Not Hispanic or Latino | 1 (1.6%) | 62 (39.0%) |
| **Race, n (%)** |  |  |
| Asian | 0 (0%) | 2 (1.3%) |
| Black or African American | 0 (0%) | 21 (13.2%) |
| Mestizo | 59 (96.7%) | 93 (58.5%) |
| White | 2 (3.3%) | 41 (25.8%) |
| Other | 0 (0%) | 2 (1.3%) |
| **Employment Status, n (%)** |  |  |
| Employed, full time or part-time | 29 (47.5%) | 91 (57.2%) |
| Retired | 1 (1.6%) | 0 (0%) |
| Other^1^ | 31 (50.8%) | 22 (13.8%) |
| Missing | 0 (0%) | 46 (28.9%) |
| **Military Status, n (%)** |  |  |
| Never in the military | 61 (100.0%) | 92 (57.9%) |
| Active/Retired/Reserves | 0 (0%) | 18 (11.3%) |
| Other | 0 (0%) | 1 (0.6%) |
| Missing | 0 (0%) | 48 (30.2%) |
| **Highest Level of Education** |  |  |
| Elementary/primary school | 11 (18.0%) | 4 (2.5%) |
| Secondary/high school or less | 26 (42.6%) | 33 (20.8%) |
| Some college | 4 (6.6%) | 11 (6.9%) |
| College degree or more | 15 (24.6%) | 62 (39.0%) |
| Other | 5 (8.2%) | 49 (30.8%) |
| **Current Treatments, n (%)** |  |  |
| None | 18 (29.5%) | 79 (49.7%) |
| Oseltamivir (Tamiflu) | 13 (21.3%) | 8 (5.0%) |
| Amantadine (Symmetrel) | 0 (0%) | 6 (3.8%) |
| Other | 34 (55.7%) | 74 (46.5%) |
| Acetaminophen | 8 (13.1%) | 32 (20.1%) |
| Antibiotic | 15 (24.6%) | 16 (10.1%) |
| Antiflu | 1 (1.6%) | 9 (5.7%) |
| Antihistamine | 1 (1.6%) | 10 (6.3%) |
| Aspirin | 1 (1.6%) | 1 (0.6%) |
| Cough suppressant or expectorant | 0 (0%) | 3 (1.9%) |
| Decongestant | 0 (0%) | 1 (0.6%) |
| Inhaled corticosteroid | 2 (3.3%) | 0 (0%) |
| Mucolytic | 2 (3.3%) | 2 (1.3%) |
| NSAID | 1 (1.6%) | 14 (8.8%) |
| OTC: symptom relief | 1 (1.6%) | 2 (1.3%) |
| Short-acting beta agonist | 1 (1.6%) | 0 (0%) |
| **Co-morbidities^2^, n (%)** |  |  |
| None | 15 (24.6%) | 96 (60.4%) |
| Asthma | 9 (14.8%) | 13 (8.2%) |
| Chronic Obstructive Pulmonary Disease (COPD) | 3 (4.9%) | 0 (0%) |
| Osteoporosis | 4 (6.6%) | 0 (0%) |
| Depression | 9 (14.8%) | 6 (3.8%) |
| Hypertension | 17 (27.9%) | 13 (8.2%) |
| Raised cholesterol | 4 (6.6%) | 15 (9.4%) |
| Stomach ulcers | 2 (3.3%) | 3 (1.9%) |
| Heart attack/angina | 2 (3.3%) | 3 (1.9%) |
| Diabetes | 14 (23.0%) | 9 (5.7%) |
| Kidney disease | 10 (16.4%) | 2 (1.3%) |
| Lung disease | 8 (13.1%) | 0 (0%) |
| Tuberculosis | 1 (1.6%) | 2 (1.3%) |
| Other | 25 (41.0%) | 27 (17.0%) |

^1^Other includes homemaker, student, unemployed, disabled, and other

^2^Not mutually exclusive

Table B in S1 File. FLU-PRO Domain and Total Score Descriptive Statistics in Non-Hospitalized Patients - Day 1

|  | N | Mean ± SD | Range | Median | Mode | Floor n (%) | Ceiling n (%) | Missing n (%) |
| --- | --- | --- | --- | --- | --- | --- | --- | --- |
| Nose | 157 | 1.8 ± 1.0 | 0.0-3.8 | 1.8 | 3.0 | 6 (3.8%) | 0 (0.0%) | 2 (1.3%) |
| Throat | 157 | 1.8 ± 1.2 | 0.0-4.0 | 1.7 | 0.3 | 7 (4.5%) | 4 (2.5%) | 2 (1.3%) |
| Eyes | 157 | 1.1 ± 1.0 | 0.0-4.0 | 1.0 | 0.0 | 32 (20.4%) | 2 (1.3%) | 2 (1.3%) |
| Chest/Respiratory | 157 | 1.4 ± 0.8 | 0.0-3.7 | 1.3 | 1.3 | 7 (4.5%) | 0 (0.0%) | 2 (1.3%) |
| Gastrointestinal | 158 | 0.5 ± 0.7 | 0.0-3.5 | 0.3 | 0.0 | 74 (46.8%) | 0 (0.0%) | 1 (0.6%) |
| Body/Systemic | 158 | 1.6 ± 0.9 | 0.1-3.6 | 1.5 | 0.7 | 0 (0.0%) | 0 (0.0%) | 1 (0.6%) |
| Total Score | 157 | 1.4 ± 0.6 | 0.0-3.1 | 1.4 | 1.7 | 0 (0.0%) | 0 (0.0%) | 2 (1.3%) |

Table C in S1 File. FLU-PRO Domain and Total Score Descriptive Statistics in Hospitalized Patients - Day 1

|  | N | Mean ± SD | Range | Median | Mode | Floor n (%) | Ceiling n (%) | Missing n (%) |
| --- | --- | --- | --- | --- | --- | --- | --- | --- |
| Nose | 61 | 0.6 ± 0.5 | 0.0-2.3 | 0.5 | 0.0 | 14 (23.0%) | 0 (0.0%) | 0 (0.0%) |
| Throat | 61 | 0.8 ± 0.9 | 0.0-4.0 | 0.7 | 0.0 | 19 (31.1%) | 1 (1.6%) | 0 (0.0%) |
| Eyes | 61 | 0.5 ± 0.8 | 0.0-4.0 | 0.3 | 0.0 | 29 (47.5%) | 1 (1.6%) | 0 (0.0%) |
| Chest/Respiratory | 61 | 1.5 ± 0.8 | 0.0-3.6 | 1.4 | 1.3 | 2 (3.3%) | 0 (0.0%) | 0 (0.0%) |
| Gastrointestinal | 61 | 0.4 ± 0.6 | 0.0-2.3 | 0.3 | 0.0 | 29 (47.5%) | 0 (0.0%) | 0 (0.0%) |
| Body/Systemic | 61 | 1.0 ± 0.8 | 0.0-3.4 | 0.7 | 0.7 | 2 (3.3%) | 0 (0.0%) | 0 (0.0%) |
| Total Score | 61 | 0.9 ± 0.5 | 0.2-2.5 | 0.8 | 0.8 | 0 (0.0%) | 0 (0.0%) | 0 (0.0%) |

Table D in S1 File. Two-day Reliability of FLU-PRO - Days 1 to Day 7 in Non-Hospitalized Patients

| FLU-PRO Scores | N^1^ | Day X Mean (SD) | Day Y Mean (SD) | Mean Difference (SD)^2^ | T Statistic | p value | Effect Size | ICC |
| --- | --- | --- | --- | --- | --- | --- | --- | --- |
| **Day 1 to Day 2** |  |  |  |  |  |  |  |  |
| Nose | 45 | 1.9 (1.0) | 1.8 (0.9) | 0.1 (0.7) | 1.14 | 0.2590 | 0.11 | 0.77 |
| Throat | 45 | 1.8 (1.1) | 1.5 (1.1) | 0.3 (1.0) | 2.01 | 0.0509 | 0.25 | 0.62 |
| Eyes | 45 | 1.1 (1.0) | 1.1 (0.9) | 0.1 (0.9) | 0.52 | 0.6034 | 0.06 | 0.63 |
| Chest/Respiratory | 45 | 1.4 (0.8) | 1.4 (0.7) | 0.0 (0.6) | 0.26 | 0.7998 | 0.03 | 0.73 |
| Gastrointestinal | 45 | 0.5 (0.7) | 0.4 (0.5) | 0.1 (0.6) | 1.09 | 0.2799 | 0.14 | 0.46 |
| Body/Systemic | 45 | 1.5 (0.6) | 1.2 (0.7) | 0.3 (0.6) | 2.67 | 0.0105 | 0.39 | 0.54 |
| Total Score | 45 | 1.4 (0.5) | 1.2 (0.5) | 0.2 (0.4) | 2.31 | 0.0256 | 0.28 | 0.63 |
| **Day 2 to Day 3** |  |  |  |  |  |  |  |  |
| Nose | 37 | 1.5 (0.8) | 1.4 (0.8) | 0.1 (0.4) | 1.38 | 0.1773 | 0.12 | 0.87 |
| Throat | 37 | 1.5 (1.1) | 1.3 (0.9) | 0.2 (0.7) | 1.99 | 0.0540 | 0.21 | 0.75 |
| Eyes | 37 | 0.9 (0.8) | 0.8 (0.9) | 0.1 (0.7) | 0.86 | 0.3963 | 0.12 | 0.69 |
| Chest/Respiratory | 37 | 1.3 (0.8) | 1.4 (0.8) | -0.1 (0.6) | -1.07 | 0.2940 | 0.13 | 0.75 |
| Gastrointestinal | 37 | 0.4 (0.7) | 0.4 (0.5) | 0.1 (0.6) | 0.65 | 0.5179 | 0.08 | 0.60 |
| Body/Systemic | 37 | 1.2 (0.8) | 1.0 (0.7) | 0.1 (0.5) | 1.76 | 0.0870 | 0.16 | 0.82 |
| Total Score | 37 | 1.2 (0.7) | 1.1 (0.6) | 0.1 (0.3) | 1.60 | 0.1187 | 0.11 | 0.90 |
| **Day 3 to Day 4** |  |  |  |  |  |  |  |  |
| Nose | 33 | 1.5 (0.8) | 1.5 (0.8) | 0.0 (0.6) | 0.23 | 0.8215 | 0.03 | 0.73 |
| Throat | 33 | 1.5 (1.1) | 1.2 (1.0) | 0.3 (0.6) | 3.23 | 0.0028 | 0.30 | 0.81 |
| Eyes | 33 | 0.8 (1.0) | 0.6 (0.7) | 0.2 (0.8) | 1.27 | 0.2125 | 0.19 | 0.54 |
| Chest/Respiratory | 33 | 1.4 (0.8) | 1.4 (0.8) | 0.0 (0.5) | 0.52 | 0.6078 | 0.05 | 0.81 |
| Gastrointestinal | 33 | 0.2 (0.4) | 0.2 (0.5) | 0.0 (0.4) | 0.00 | 1.0000 | 0.00 | 0.46 |
| Body/Systemic | 33 | 1.0 (0.8) | 0.8 (0.5) | 0.2 (0.5) | 1.84 | 0.0755 | 0.22 | 0.65 |
| Total Score | 33 | 1.1 (0.6) | 1.0 (0.5) | 0.1 (0.4) | 1.76 | 0.0876 | 0.20 | 0.73 |
| **Day 4 to Day 5** |  |  |  |  |  |  |  |  |
| Nose | 23 | 1.2 (0.8) | 1.0 (0.7) | 0.2 (0.4) | 2.05 | 0.0525 | 0.20 | 0.85 |
| Throat | 23 | 0.9 (0.9) | 0.8 (0.9) | 0.0 (0.6) | 0.38 | 0.7099 | 0.05 | 0.83 |
| Eyes | 23 | 0.7 (0.9) | 0.4 (0.7) | 0.3 (0.5) | 2.59 | 0.0166 | 0.32 | 0.75 |
| Chest/Respiratory | 23 | 1.1 (0.7) | 1.0 (0.7) | 0.1 (0.3) | 1.95 | 0.0636 | 0.15 | 0.93 |
| Gastrointestinal | 23 | 0.2 (0.4) | 0.3 (0.5) | -0.1 (0.4) | -0.83 | 0.4175 | 0.15 | 0.68 |
| Body/Systemic | 23 | 0.8 (0.7) | 0.7 (0.8) | 0.0 (0.3) | 0.23 | 0.8220 | 0.02 | 0.89 |
| Total Score | 23 | 0.8 (0.5) | 0.8 (0.6) | 0.1 (0.2) | 1.70 | 0.1031 | 0.13 | 0.93 |
| **Day 5 to Day 6** |  |  |  |  |  |  |  |  |
| Nose | 25 | 0.9 (0.6) | 0.9 (0.6) | -0.0 (0.4) | -0.13 | 0.8995 | 0.02 | 0.77 |
| Throat | 25 | 0.9 (1.1) | 0.9 (1.0) | 0.1 (0.2) | 1.07 | 0.2943 | 0.05 | 0.97 |
| Eyes | 25 | 0.5 (0.8) | 0.4 (0.7) | 0.1 (0.6) | 0.48 | 0.6322 | 0.07 | 0.71 |
| Chest/Respiratory | 25 | 1.1 (0.8) | 1.1 (0.7) | -0.0 (0.4) | -0.00 | 1.0000 | 0.00 | 0.84 |
| Gastrointestinal | 25 | 0.3 (0.5) | 0.2 (0.5) | 0.1 (0.4) | 0.67 | 0.5113 | 0.11 | 0.68 |
| Body/Systemic | 25 | 0.8 (0.7) | 0.6 (0.6) | 0.1 (0.3) | 2.21 | 0.0366 | 0.18 | 0.90 |
| Total Score | 25 | 0.8 (0.6) | 0.7 (0.5) | 0.1 (0.2) | 1.18 | 0.2489 | 0.10 | 0.90 |
| **Day 6 to Day 7** |  |  |  |  |  |  |  |  |
| Nose | 22 | 0.9 (0.7) | 0.9 (0.7) | -0.0 (0.4) | -0.53 | 0.6046 | 0.06 | 0.83 |
| Throat | 22 | 0.9 (1.0) | 0.9 (1.0) | -0.0 (0.5) | -0.40 | 0.6902 | 0.05 | 0.86 |
| Eyes | 22 | 0.2 (0.3) | 0.2 (0.3) | 0.0 (0.2) | 1.14 | 0.2664 | 0.15 | 0.78 |
| Chest/Respiratory | 22 | 1.0 (0.6) | 1.1 (0.6) | -0.1 (0.5) | -1.15 | 0.2631 | 0.20 | 0.67 |
| Gastrointestinal | 22 | 0.1 (0.3) | 0.1 (0.3) | -0.0 (0.2) | -0.62 | 0.5396 | 0.08 | 0.80 |
| Body/Systemic | 22 | 0.5 (0.5) | 0.3 (0.4) | 0.1 (0.3) | 1.80 | 0.0862 | 0.24 | 0.72 |
| Total Score | 22 | 0.6 (0.4) | 0.6 (0.3) | 0.0 (0.2) | 0.15 | 0.8788 | 0.02 | 0.83 |

^1^Number of study participants with no change in flu symptom at day Y.

^2^Mean difference = average Day X FLU-PRO score - average Day Y FLU-PRO score (ex. Day 1 score - Day 2 score); p value from paired t-test.

Table E in S1 File. Two-day Reliability of FLU-PRO - Days 1 to Day 7 in Hospitalized Patients

| FLU-PRO Scores | N^1^ | Day X Mean (SD) | Day Y Mean (SD) | Mean Difference (SD)^2^ | T Statistic | p value | Effect Size | ICC |
| --- | --- | --- | --- | --- | --- | --- | --- | --- |
| **Day 1 to Day 2** |  |  |  |  |  |  |  |  |
| Nose | 5 | 0.7 (0.5) | 0.8 (0.7) | -0.1 (0.6) | -0.19 | 0.8605 | 0.10 | 0.52 |
| Throat | 5 | 0.6 (0.8) | 0.2 (0.4) | 0.4 (0.7) | 1.24 | 0.2835 | 0.48 | 0.39 |
| Eyes | 5 | 0.2 (0.3) | 0.2 (0.2) | 0.0 (0.2) | 0.00 | 1.0000 | 0.00 | 0.60 |
| Chest/Respiratory | 5 | 1.3 (0.3) | 1.1 (0.2) | 0.2 (0.1) | 3.50 | 0.0249 | 0.68 | 0.69 |
| Gastrointestinal | 5 | 0.2 (0.3) | 0.0 (0.0) | 0.2 (0.3) | 1.37 | 0.2420 | 0.61 | 0.00 |
| Body/Systemic | 5 | 1.1 (1.1) | 0.8 (0.5) | 0.3 (1.1) | 0.64 | 0.5583 | 0.30 | 0.08 |
| Total Score | 5 | 0.8 (0.4) | 0.6 (0.2) | 0.2 (0.4) | 1.10 | 0.3349 | 0.56 | -0.09 |
| **Day 2 to Day 3** |  |  |  |  |  |  |  |  |
| Nose | 7 | 0.7 (0.8) | 0.9 (0.5) | -0.1 (0.6) | -0.66 | 0.5352 | 0.17 | 0.66 |
| Throat | 7 | 0.8 (1.4) | 0.9 (0.9) | -0.1 (1.0) | -0.25 | 0.8085 | 0.07 | 0.68 |
| Eyes | 7 | 0.5 (0.5) | 0.5 (0.5) | 0.0 (0.3) | 0.00 | 1.0000 | 0.00 | 0.82 |
| Chest/Respiratory | 7 | 1.6 (0.8) | 1.7 (0.9) | -0.1 (0.3) | -0.85 | 0.4262 | 0.12 | 0.94 |
| Gastrointestinal | 8 | 0.0 (0.1) | 0.5 (0.4) | -0.5 (0.3) | -3.91 | 0.0058 | 5.30 | 0.10 |
| Body/Systemic | 7 | 0.9 (0.9) | 0.8 (0.7) | 0.1 (0.4) | 0.84 | 0.4312 | 0.16 | 0.85 |
| Total Score | 7 | 0.9 (0.6) | 0.9 (0.5) | -0.0 (0.2) | -0.79 | 0.4590 | 0.09 | 0.96 |
| **Day 3 to Day 4** |  |  |  |  |  |  |  |  |
| Nose | 8 | 0.2 (0.4) | 0.3 (0.4) | -0.1 (0.3) | -0.89 | 0.4015 | 0.26 | 0.70 |
| Throat | 8 | 0.3 (0.7) | 0.4 (0.5) | -0.0 (0.8) | -0.14 | 0.8929 | 0.06 | 0.05 |
| Eyes | 8 | 0.3 (0.3) | 0.2 (0.3) | 0.1 (0.2) | 1.43 | 0.1970 | 0.41 | 0.65 |
| Chest/Respiratory | 8 | 1.3 (0.6) | 1.4 (0.7) | -0.1 (0.4) | -0.53 | 0.6131 | 0.12 | 0.84 |
| Gastrointestinal | 9 | 0.2 (0.3) | 0.1 (0.2) | 0.1 (0.2) | 1.51 | 0.1690 | 0.42 | 0.56 |
| Body/Systemic | 9 | 0.3 (0.3) | 0.6 (0.6) | -0.3 (0.5) | -1.83 | 0.1044 | 0.97 | 0.36 |
| Total Score | 8 | 0.5 (0.2) | 0.6 (0.3) | -0.1 (0.3) | -1.28 | 0.2425 | 0.58 | 0.48 |
| **Day 4 to Day 5** |  |  |  |  |  |  |  |  |
| Nose | 7 | 0.2 (0.4) | 0.4 (0.3) | -0.1 (0.4) | -1.00 | 0.3559 | 0.39 | 0.33 |
| Throat | 7 | 0.1 (0.2) | 0.5 (1.1) | -0.4 (1.0) | -0.98 | 0.3639 | 2.14 | 0.16 |
| Eyes | 7 | 0.0 (0.1) | 0.1 (0.2) | -0.0 (0.1) | -1.00 | 0.3559 | 0.38 | 0.62 |
| Chest/Respiratory | 7 | 0.9 (0.4) | 0.9 (0.5) | 0.0 (0.6) | 0.00 | 1.0000 | 0.00 | 0.23 |
| Gastrointestinal | 8 | 0.2 (0.2) | 0.2 (0.2) | -0.0 (0.2) | -0.55 | 0.5983 | 0.14 | 0.76 |
| Body/Systemic | 8 | 0.3 (0.3) | 0.6 (0.4) | -0.2 (0.5) | -1.37 | 0.2126 | 0.69 | 0.23 |
| Total Score | 7 | 0.4 (0.2) | 0.5 (0.3) | -0.2 (0.3) | -1.26 | 0.2540 | 0.89 | 0.25 |
| **Day 5 to Day 6** |  |  |  |  |  |  |  |  |
| Nose | 7 | 0.4 (0.3) | 0.4 (0.3) | -0.0 (0.4) | -0.21 | 0.8382 | 0.11 | 0.14 |
| Throat | 7 | 0.0 (0.1) | 0.0 (0.1) | 0.0 (0.0) |  |  | 0.00 | 1.00 |
| Eyes | 7 | 0.0 (0.1) | 0.1 (0.3) | -0.1 (0.3) | -1.00 | 0.3559 | 0.76 | 0.25 |
| Chest/Respiratory | 7 | 0.9 (0.5) | 0.8 (0.9) | 0.0 (0.7) | 0.16 | 0.8797 | 0.09 | 0.57 |
| Gastrointestinal | 7 | 0.1 (0.2) | 0.1 (0.2) | 0.0 (0.0) |  |  | 0.00 | 1.00 |
| Body/Systemic | 7 | 0.5 (0.5) | 0.4 (0.5) | 0.1 (0.4) | 0.39 | 0.7066 | 0.14 | 0.60 |
| Total Score | 7 | 0.4 (0.3) | 0.4 (0.4) | 0.0 (0.3) | 0.15 | 0.8822 | 0.07 | 0.59 |
| **Day 6 to Day 7** |  |  |  |  |  |  |  |  |
| Nose | 5 | 0.5 (0.4) | 0.6 (0.5) | -0.1 (0.3) | -0.67 | 0.5415 | 0.28 | 0.76 |
| Throat | 5 | 0.2 (0.3) | 0.3 (0.3) | -0.1 (0.3) | -1.00 | 0.3739 | 0.45 | 0.56 |
| Eyes | 5 | 0.0 (0.0) | 0.3 (0.3) | -0.3 (0.3) | -2.14 | 0.0993 |  | 0.00 |
| Chest/Respiratory | 5 | 1.2 (1.0) | 1.1 (0.5) | 0.1 (0.5) | 0.38 | 0.7267 | 0.08 | 0.83 |
| Gastrointestinal | 5 | 0.1 (0.2) | 0.1 (0.2) | 0.0 (0.0) |  |  | 0.00 | 1.00 |
| Body/Systemic | 5 | 0.4 (0.3) | 0.3 (0.2) | 0.1 (0.1) | 2.67 | 0.0560 | 0.42 | 0.83 |
| Total Score | 5 | 0.5 (0.4) | 0.5 (0.3) | 0.0 (0.1) | 0.38 | 0.7215 | 0.05 | 0.96 |

^1^Number of study participants with no change in flu symptom at day Y. No data available for Day 4 to Day 5.

^2^Mean difference = average Day X FLU-PRO score - average Day Y FLU-PRO score (ex. Day 1 score - Day 2 score); p value from paired t-test.

Table F in S1 File. Construct Validity: FLU-PRO Scale Correlations with Other PRO Measures at Day 1 in Non-Hospitalized Patients

|  | Domains and Total Score^1^ | | | | | | |
| --- | --- | --- | --- | --- | --- | --- | --- |
| **Day** | **Nose** | **Throat** | **Eyes** | **Chest/ Respiratory** | **Gastrointestinal** | **Body/ Systemic** | **Total Score** |
| **Day 1** |  |  |  |  |  |  |  |
| Patient Global Rating of Flu Severity | 0.46^***^ | 0.52^***^ | 0.42^***^ | 0.38^***^ | 0.14 | 0.59^***^ | 0.67^***^ |
| Patient Global Rating of Physical Health | -0.28^**^ | -0.38^***^ | -0.27^**^ | -0.34^***^ | -0.24^*^ | -0.47^***^ | -0.52^***^ |
| Patient Global Assessment of Interference in Daily Activities | 0.39^***^ | 0.36^***^ | 0.40^***^ | 0.34^***^ | 0.30^**^ | 0.63^***^ | 0.65^***^ |

^1^Spearman correlation coefficients: ^***^p<0.0001, ^**^p<0.001, ^*^p<0.05

Table G in S1 File. Construct Validity: FLU-PRO Scale Correlations with Other PRO Measures at Day 1 in Hospitalized Patients

|  | Domains and Total Score^1^ | | | | | | |
| --- | --- | --- | --- | --- | --- | --- | --- |
| **Day** | **Nose** | **Throat** | **Eyes** | **Chest/ Respiratory** | **Gastrointestinal** | **Body/ Systemic** | **Total Score** |
| **Day 1** |  |  |  |  |  |  |  |
| Patient Global Rating of Flu Severity | 0.01 | 0.08 | 0.45^**^ | 0.11 | 0.32^*^ | 0.53^***^ | 0.50^***^ |
| Patient Global Rating of Physical Health | -0.14 | 0.07 | -0.13 | -0.18 | -0.09 | -0.17 | -0.14 |
| Patient Global Assessment of Interference in Daily Activities | 0.05 | 0.09 | -0.01 | 0.07 | 0.03 | 0.32^*^ | 0.28^*^ |

^1^Spearman correlation coefficients: ^***^p<0.0001, ^**^p<0.001, ^*^p<0.05

Table H in S1 File. Known-Groups Validity: FLU-PRO Scores by Patient Global Rating of Disease Severity, Day 1 in Non-Hospitalized Patients

|  | Patient Global Rating of Flu Severity | | | | | |  | |
| --- | --- | --- | --- | --- | --- | --- | --- | --- |
|  | **No/Mild** | |  | | **Severe and** | |  | |
|  | **Symptoms** | | **Moderate** | | **Very Severe** | |  | |
| **Scale** | **N** | **Mean (SD)** | **N** | **Mean (SD)** | **N** | **Mean (SD)** | **F Value (p value)^1^** | **Pairwise Comparisons^2^** |
| Nose | 48 | 1.15 (0.79) | 58 | 1.98 (0.85) | 51 | 2.25 (0.93) | 22.3*** | 1***,2*** |
| Throat | 48 | 1.14 (1.04) | 58 | 1.77 (0.94) | 51 | 2.59 (1.06) | 25.9*** | 1**,2***,3*** |
| Eyes | 48 | 0.51 (0.61) | 58 | 1.13 (0.82) | 51 | 1.54 (1.15) | 16.9*** | 1**,2*** |
| Chest/Respiratory | 48 | 0.97 (0.72) | 58 | 1.45 (0.72) | 51 | 1.76 (0.89) | 12.8*** | 1**,2*** |
| Gastrointestinal | 48 | 0.35 (0.69) | 58 | 0.41 (0.57) | 52 | 0.66 (0.88) | 2.7 |  |
| Body/Systemic | 48 | 0.94 (0.69) | 58 | 1.50 (0.70) | 52 | 2.17 (0.72) | 38.1*** | 1***,2***,3*** |
| Total Score | 48 | 0.88 (0.50) | 58 | 1.40 (0.46) | 51 | 1.88 (0.45) | 56.4*** | 1***,2***,3*** |

^1^p values are: *<0.05, **<0.01, ***<0.001.

^2^Pairwise comparisons between means will be performed using Scheffe's test adjusting for multiple comparisons: 1=No/Mild symptoms vs Moderate, 2=No/Mild symptoms vs Severe and Very Severe, and 3=Moderate symptoms vs Severe and Very Severe.

Table I in S1 File. Known-Groups Validity: FLU-PRO Scores by Patient Global Rating of Disease Severity, Day 1 in Hospitalized Patients

|  | Patient Global Rating of Flu Severity | | | | | |  | |
| --- | --- | --- | --- | --- | --- | --- | --- | --- |
|  | **No/Mild** | |  | | **Severe and** | |  | |
|  | **Symptoms** | | **Moderate** | | **Very Severe** | |  | |
| **Scale** | **N** | **Mean (SD)** | **N** | **Mean (SD)** | **N** | **Mean (SD)** | **F Value (p value)^1^** | **Pairwise Comparisons^2^** |
| Nose | 33 | 0.52 (0.45) | 19 | 0.63 (0.59) | 9 | 0.58 (0.77) | 0.2 |  |
| Throat | 33 | 0.73 (0.84) | 19 | 0.60 (0.49) | 9 | 1.33 (1.50) | 2.3 |  |
| Eyes | 33 | 0.16 (0.24) | 19 | 0.77 (0.88) | 9 | 1.15 (1.36) | 8.6*** | 1*,2** |
| Chest/Respiratory | 33 | 1.39 (0.67) | 19 | 1.35 (0.63) | 9 | 1.97 (1.17) | 2.4 |  |
| Gastrointestinal | 33 | 0.27 (0.46) | 19 | 0.32 (0.38) | 9 | 1.08 (0.89) | 9.0*** | 2***,3** |
| Body/Systemic | 33 | 0.67 (0.57) | 19 | 1.09 (0.65) | 9 | 2.06 (0.91) | 16.3*** | 2***,3** |
| Total Score | 33 | 0.72 (0.35) | 19 | 0.92 (0.33) | 9 | 1.58 (0.63) | 17.0*** | 2***,3*** |

^1^p values are: *<0.05, **<0.01, ***<0.001.

^2^Pairwise comparisons between means will be performed using Scheffe's test adjusting for multiple comparisons: 1=No/Mild symptoms vs Moderate, 2=No/Mild symptoms vs Severe and Very Severe, and 3=Moderate symptoms vs Severe and Very Severe.

Table J in S1 File. Responsiveness of FLU-PRO by Patient Return to Usual Health (N=119)^1^ and Usual Activities (N=67)^2^, Day 1 to Day 7 in Non-Hospitalized Patients

|  | Responders^3^ | | | Non-Responders | | |  |
| --- | --- | --- | --- | --- | --- | --- | --- |
| **Scale** | **Day 1 Mean (SD)** | **Day 7 Mean (SD)** | **Change Score LS Mean (SDerr)** | **Day 1 Mean (SD)** | **Day 7 Mean (SD)** | **Change Score LS Mean (SDerr)** | **p-value^2^** |
| **Nose** |  |  |  |  |  |  |  |
| Usual Health | 1.8 (1.0) | 0.6 (0.6) | 1.3 (0.1) | 1.9 (0.9) | 1.0 (0.7) | 0.8 (0.1) | 0.0003 |
| Usual Activities | 1.8 (1.1) | 0.8 (0.8) | 1.0 (0.1) | 1.9 (0.8) | 1.1 (0.7) | 0.8 (0.2) | 0.4609 |
| **Throat** |  |  |  |  |  |  |  |
| Usual Health | 1.6 (1.1) | 0.4 (0.6) | 1.5 (0.1) | 2.1 (1.2) | 0.9 (0.9) | 1.1 (0.1) | 0.0132 |
| Usual Activities | 2.0 (1.2) | 0.6 (0.7) | 1.5 (0.1) | 2.1 (1.2) | 1.1 (1.2) | 1.0 (0.2) | 0.0968 |
| **Eyes** |  |  |  |  |  |  |  |
| Usual Health | 1.1 (1.0) | 0.2 (0.5) | 0.9 (0.1) | 1.0 (0.9) | 0.3 (0.7) | 0.7 (0.1) | 0.1610 |
| Usual Activities | 1.1 (1.0) | 0.2 (0.5) | 0.9 (0.1) | 1.1 (1.1) | 0.5 (1.0) | 0.5 (0.2) | 0.0366 |
| **Chest/Respiratory** |  |  |  |  |  |  |  |
| Usual Health | 1.3 (0.8) | 0.6 (0.6) | 0.8 (0.1) | 1.5 (0.8) | 1.2 (0.7) | 0.3 (0.1) | <.0001 |
| Usual Activities | 1.4 (0.8) | 0.9 (0.7) | 0.6 (0.1) | 2.2 (0.8) | 1.5 (0.9) | 0.2 (0.2) | 0.1322 |
| **Gastrointestinal** |  |  |  |  |  |  |  |
| Usual Health | 0.4 (0.8) | 0.1 (0.4) | 0.3 (0.0) | 0.4 (0.6) | 0.2 (0.4) | 0.2 (0.0) | 0.0522 |
| Usual Activities | 0.4 (0.7) | 0.1 (0.2) | 0.4 (0.0) | 0.7 (0.8) | 0.3 (0.4) | 0.2 (0.1) | 0.0210 |
| **Body/Systemic** |  |  |  |  |  |  |  |
| Usual Health | 1.5 (0.8) | 0.2 (0.4) | 1.3 (0.1) | 1.6 (0.8) | 0.6 (0.6) | 1.0 (0.1) | 0.0005 |
| Usual Activities | 1.8 (0.8) | 0.4 (0.4) | 1.4 (0.1) | 1.8 (0.9) | 0.9 (0.7) | 0.8 (0.1) | 0.0001 |
| **Total Score** |  |  |  |  |  |  |  |
| Usual Health | 1.3 (0.6) | 0.4 (0.3) | 1.0 (0.1) | 1.5 (0.6) | 0.7 (0.5) | 0.7 (0.0) | <.0001 |
| Usual Activities | 1.5 (0.6) | 0.5 (0.4) | 1.0 (0.1) | 1.7 (0.6) | 1.0 (0.7) | 0.6 (0.1) | 0.0057 |

^1^Responders: N=49; Non-responders: N=70

^2^Responders: N=55; Non-responders: N=12

^3^Responders are defined as patients responding that they have returned to their usual health or usual activities at Day 7.

Table K in S1 File. Responsiveness of FLU-PRO by Patient Return to Usual Health (N=50)^1^ and Usual Activities (N=50)^2^, Day 1 to Day 7 in Hospitalized Patients

|  | Responders^3^ | | | Non-Responders | | |  |
| --- | --- | --- | --- | --- | --- | --- | --- |
| **Scale** | **Day 1 Mean (SD)** | **Day 7 Mean (SD)** | **Change Score LS Mean (SDerr)** | **Day 1 Mean (SD)** | **Day 7 Mean (SD)** | **Change Score LS Mean (SDerr)** | **p-value^2^** |
| **Nose** |  |  |  |  |  |  |  |
| Usual Health | 0.7 (0.6) | 0.1 (0.2) | 0.5 (0.1) | 0.6 (0.5) | 0.3 (0.4) | 0.3 (0.1) | 0.0898 |
| Usual Activities | 0.7 (0.6) | 0.1 (0.2) | 0.5 (0.1) | 0.5 (0.5) | 0.3 (0.4) | 0.3 (0.1) | 0.1034 |
| **Throat** |  |  |  |  |  |  |  |
| Usual Health | 0.9 (0.9) | 0.1 (0.2) | 0.7 (0.1) | 0.8 (0.9) | 0.4 (0.6) | 0.4 (0.1) | 0.0597 |
| Usual Activities | 1.0 (0.9) | 0.1 (0.3) | 0.7 (0.1) | 0.8 (1.0) | 0.4 (0.6) | 0.4 (0.1) | 0.1300 |
| **Eyes** |  |  |  |  |  |  |  |
| Usual Health | 0.4 (1.0) | 0.0 (0.1) | 0.5 (0.1) | 0.6 (0.8) | 0.1 (0.3) | 0.4 (0.0) | 0.0936 |
| Usual Activities | 0.6 (1.1) | 0.0 (0.1) | 0.5 (0.1) | 0.5 (0.8) | 0.1 (0.3) | 0.4 (0.0) | 0.1085 |
| **Chest/Respiratory** |  |  |  |  |  |  |  |
| Usual Health | 1.4 (0.6) | 0.3 (0.3) | 1.2 (0.1) | 1.5 (0.9) | 0.7 (0.6) | 0.8 (0.1) | 0.0050 |
| Usual Activities | 1.5 (0.9) | 0.4 (0.6) | 1.0 (0.1) | 1.5 (0.8) | 0.6 (0.5) | 0.8 (0.1) | 0.1653 |
| **Gastrointestinal** |  |  |  |  |  |  |  |
| Usual Health | 0.3 (0.4) | 0.0 (0.1) | 0.4 (0.1) | 0.4 (0.6) | 0.2 (0.5) | 0.3 (0.1) | 0.3724 |
| Usual Activities | 0.5 (0.6) | 0.0 (0.1) | 0.4 (0.1) | 0.4 (0.6) | 0.1 (0.5) | 0.3 (0.1) | 0.2401 |
| **Body/Systemic** |  |  |  |  |  |  |  |
| Usual Health | 0.9 (0.9) | 0.1 (0.1) | 0.9 (0.1) | 1.1 (0.8) | 0.4 (0.3) | 0.6 (0.0) | 0.0044 |
| Usual Activities | 1.1 (1.0) | 0.1 (0.2) | 0.9 (0.1) | 0.9 (0.8) | 0.3 (0.3) | 0.7 (0.0) | 0.0194 |
| **Total Score** |  |  |  |  |  |  |  |
| Usual Health | 0.9 (0.5) | 0.1 (0.1) | 0.8 (0.1) | 0.9 (0.5) | 0.4 (0.3) | 0.5 (0.0) | 0.0011 |
| Usual Activities | 1.0 (0.6) | 0.2 (0.2) | 0.8 (0.1) | 0.9 (0.5) | 0.3 (0.3) | 0.6 (0.0) | 0.0048 |

^1^Responders: N=15; Non-responders: N=35

^2^Responders: N=14; Non-responders: N=36

^3^Responders are defined as patients responding that they have returned to their usual health or usual activities at Day 7.

Table L in S1 File. FLU-PRO Domain and Total Score Descriptive Statistics by Influenza Status - Day 1

| **FLU-PRO Scales** | **Flu Positive (n=221) Mean (SD)** | **Flu Negative (n=220) Mean (SD)** | **P value** |
| --- | --- | --- | --- |
| Nose | 1.7 (1.1) | 1.5 (1.0) | 0.0271 |
| Throat | 1.4 (1.1) | 1.5 (1.2) | 0.0961 |
| Eyes | 1.0 (1.1) | 0.9 (1.0) | 0.4583 |
| Chest/Respiratory | 1.9 (0.9) | 1.4 (0.8) | <.0001 |
| Gastrointestinal | 0.7 (0.8) | 0.5 (0.7) | 0.0021 |
| Body/Systemic | 1.8 (0.9) | 1.4 (0.9) | <.0001 |
| Total Score | 1.6 (0.7) | 1.3 (0.6) | <.0001 |

Table M in S1 File. 2-Way ANOVA of FLU-PRO Domain and Total Score by Influenza and Hospitalization Status - Day 1

|  | **Hospitalized** | | | **Non-Hospitalized** | | |
| --- | --- | --- | --- | --- | --- | --- |
| **FLU-PRO Scales** | **Flu Positive (n=53) LS Mean (SE)** | **Flu Negative (n=61) LS Mean (SE)** | **P value^1^** | **Flu Positive (n=168) LS Mean (SE)** | **Flu Negative (n=159) LS Mean (SE)** | **P value^1^** |
| Nose | 1.2 (0.13) | 0.6 (0.12) | 0.0060 | 1.8 (0.07) | 1.8 (0.08) | 0.9963 |
| Throat | 1.1 (0.15) | 0.8 (0.14) | 0.5601 | 1.5 (0.09) | 1.8 (0.09) | 0.0174 |
| Eyes | 1.0 (0.14) | 0.5 (0.13) | 0.0584 | 1.0 (0.08) | 1.1 (0.08) | 0.8561 |
| Chest/Respiratory | 1.9 (0.12) | 1.5 (0.11) | 0.0703 | 1.8 (0.07) | 1.4 (0.07) | 0.0001 |
| Gastrointestinal | 0.8 (0.11) | 0.4 (0.10) | 0.0441 | 0.6 (0.06) | 0.5 (0.06) | 0.2868 |
| Body/Systemic | 1.6 (0.12) | 1.0 (0.11) | 0.0120 | 1.9 (0.07) | 1.6 (0.07) | 0.0020 |
| Total Score | 1.4 (0.09) | 0.9 (0.08) | 0.0010 | 1.6 (0.05) | 1.4 (0.05) | 0.0419 |

^1^From ANOVA with Scheffe's post-hoc adjustment.

Figure A in S1 File. FLU-PRO Domain and Total Score by Diary Days 1 to 14: Non-Hospitalized Influenza-Negative Patients


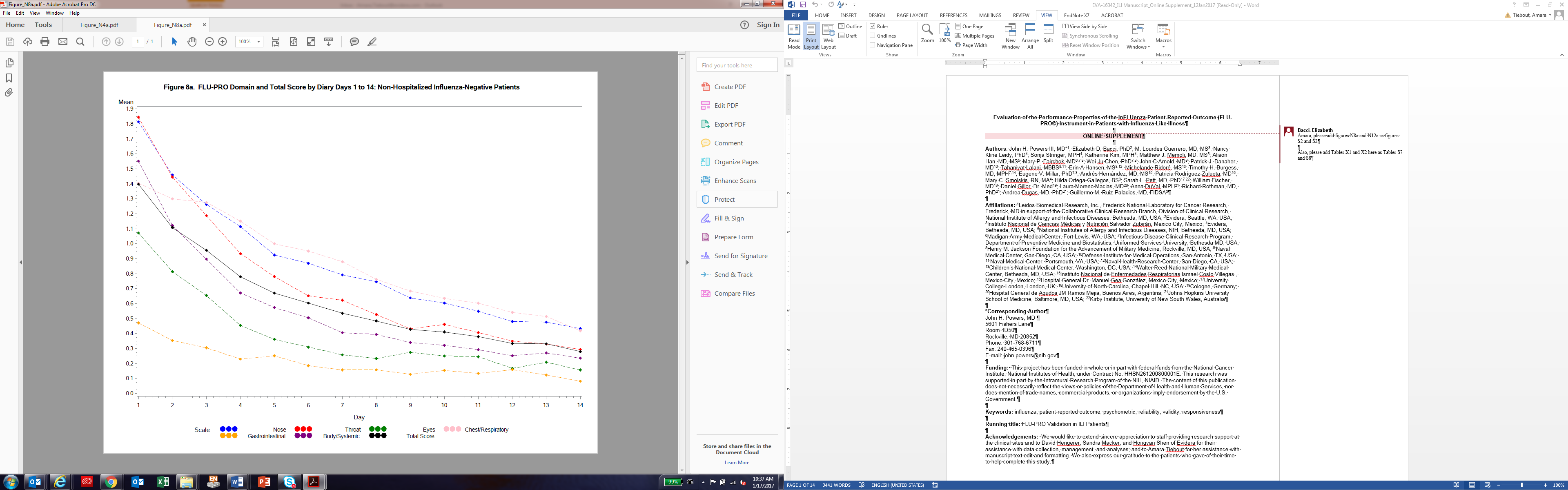


Figure B in S1 File. FLU-PRO Domain and Total Score by Diary Days 1 to 14: Hospitalized Influenza-Negative Patients


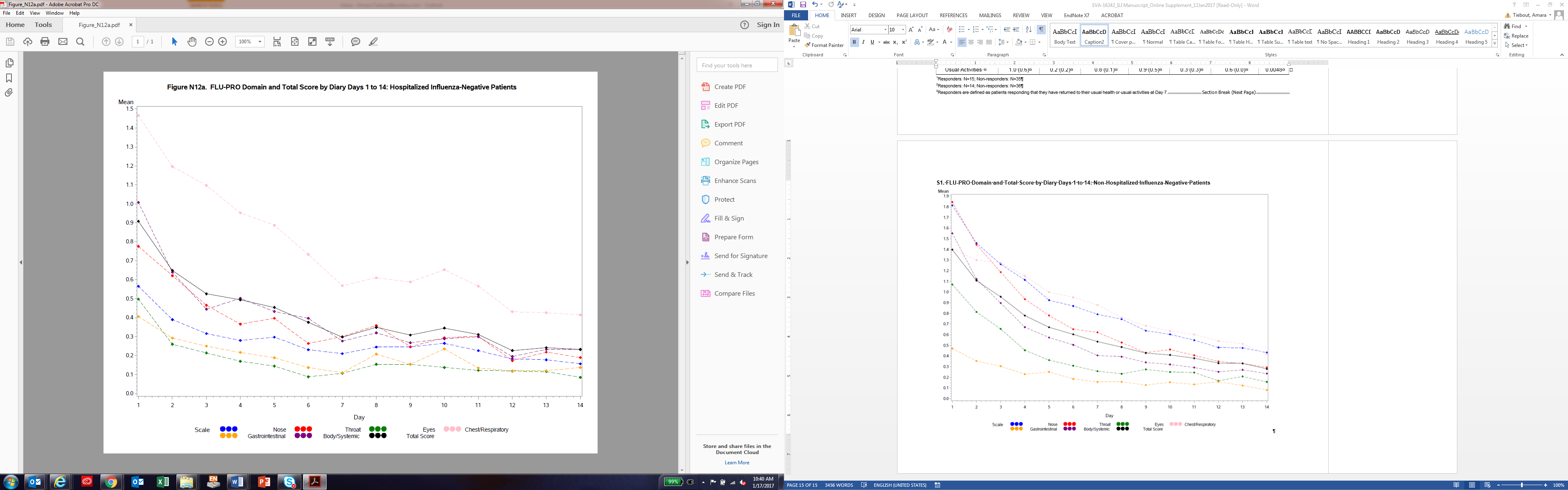


Figure C in S1 File. FLU-PRO Domain and Total Score Descriptive Statistics - Day 1

Figure D in S1 File. FLU-PRO Domain and Total Score Descriptive Statistics in Non-Hospitalized Patients - Day 1

Figure E in S1 File. FLU-PRO Domain and Total Score Descriptive Statistics in Hospitalized Patients - Day 1

Figure F in S1 File. Patient Global Rating of Flu Severity- Day 1 to 14
